# Supplementary material for: Thermodynamics shapes the in vivo enzyme burden of glycolytic pathways
Source: mBio. 2025 Sep 16;16(10):e01837-25. doi: 10.1128/mbio.01837-25 (PMC12506098; doi:10.1128/mbio.01837-25)
Supplement: Figure S1 — Correlation between AQUA quantitation and iBAQ values. [file mbio.01837-25-s0001.pdf]

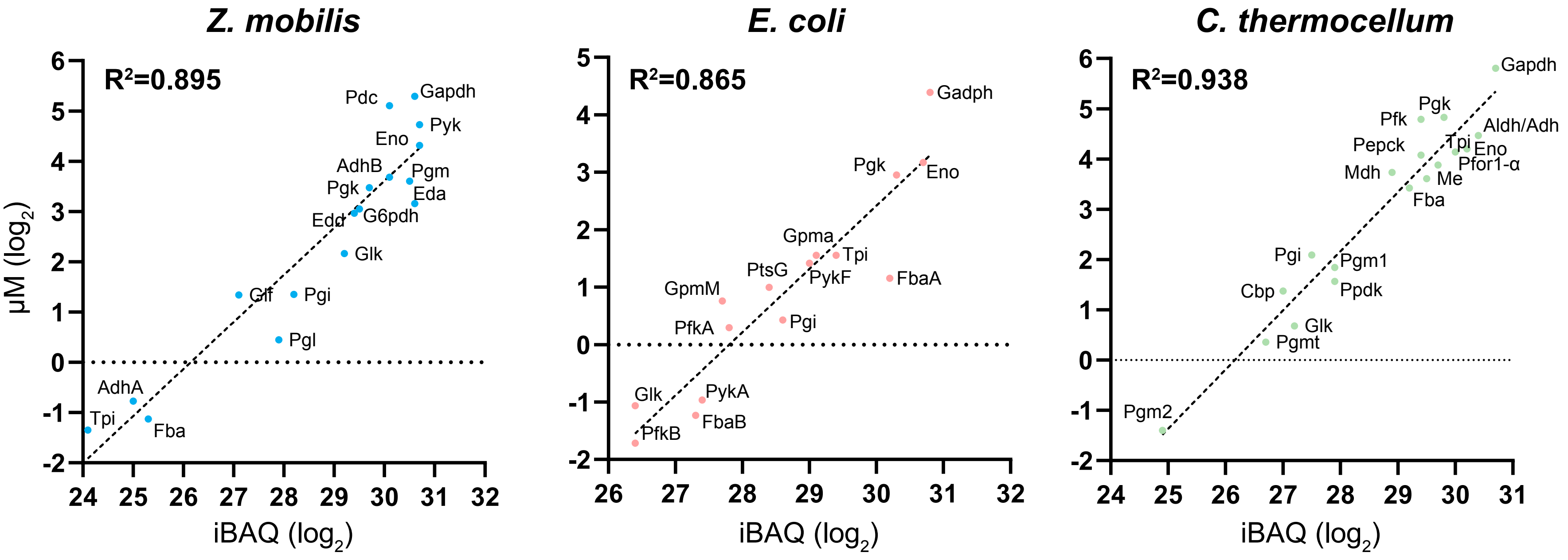

Figure S1. Correlation between AQUA quantitation and iBAQ values

Linear regression analyses between log2 transformed absolute quantification (AQUA) values (y-axis) versus intensity-based absolute quantification (iBAQ) values (x-axis) for each bacterium. Pearson's square correlation coefficients (R2) are displayed on all plots. Abbreviations: glucose facilitated diffusion protein (Glf), glucokinase (Glk), glucose 6-phosphate dehydrogenase (G6pdh), phosphogluconolactonase (Pgl), 6-phosphogluconate dehydratase (Edd), phosphoglucose isomerase (Pgi), 2-keto-3-deoxy-6-phosphogluconate aldolase (Eda), fructose 1,6-bisphosphate aldolase (Fba, FbaA, FbaB), triose phosphate isomerase (Tpi), glyceraldehyde 3-phosphate dehydrogenase (Gapdh), phosphoglycerate kinase (Pgk), phosphoglycerate mutase (Pgm, GpmM, GpmA, PGM1, PGM2), enolase (Eno), pyruvate kinase (Pyk, PykA, PykF), pyruvate decarboxylase (Pdc), alcohol dehydrogenase (AdhA, AdhB), PTS system glucose-specific EIICB component (PtsG), phosphofructokinase (PfkA, PfkB, Pfk), cellobiose phosphorylase (Cbp), phosphoglucomutase (Pgmt), pyruvate phosphate dikinase (Ppdk), phosphoenolpyruvate carboxykinase (Pepck), malate dehydrogenase (Mdh), malic enzyme (Me), pyruvate ferredoxin oxidoreductase I alpha domain (PFOR1-α), bifunctional acetaldehyde/alcohol dehydrogenase (Aldh/ Adh).
